# Research on depth measurement calibration of light field camera based on Gaussian fitting

**DOI:** 10.1038/s41598-024-59479-5

**Published:** 2024-04-16

**Authors:** Miao Yang

**Affiliations:** 1 School of Energy and Power Engineering, University of Shanghai for Science and Technology, Shanghai, 200093 China; 2 Shanghai Key Laboratory of Multiphase Flow and Heat Transfer in Power Engineering, Shanghai, 200093 China

**Keywords:** Image rendering, Gaussian fitting, Depth calibration, Depth resolution, Engineering, Optics and photonics

## Abstract

Optical field imaging technology does not require a complicated optical path layout and thus reduces hardware costs. Given that only one single exposure of a single camera can obtain three-dimensional information, this paper proposes an improved calibration method for depth measurement based on the theoretical model of optical field imaging. Specifically, the calibration time can be reduced since the Gaussian fitting can reduce the number of refocused images used to obtain the optimal refocusing coefficient calibration. Moreover, the proposed method achieves the same effect as the multiple refocusing calibration strategy but requires less image processing time during calibration. At the same time, this method's depth resolution is analyzed in detail.

## Introduction

In recent years, light field imaging technology has attracted significant interest from various fields due to its advantage in obtaining three-dimensional spatial information of objects in a single shot^1^. Light field imaging uses a light field camera to capture the four-dimensional light field information of the measurement space, namely the spatial and angle information^2^. The difference from the traditional two-dimensional camera is that a microlens array is added between the main lens and the sensor^3^. Given that during the measurement process, the accuracy of the camera depth calibration directly affects the accuracy of the entire measurement system, this paper further studies the depth calibration of the light field camera. The existing methods for calibrating the depth values of light fields can be divided into traditional and deep learning-based. Traditional methods are generally based on digital refocusing parameters or the optimal slope of linear structures in epipolar-plane images (EPI). The depth estimation algorithm based on digital refocusing parameters is a method of calculating a series of images focused at different positions (i.e., refocused images), and then obtaining the position information of the region based on a series of refocused image information at different positions. Lin et al.^4^ designed a matching term for depth estimation based on the symmetry of the focusing sequence, based on the principle that the offset on both sides of the true depth direction has consistent color. Tao et al.^5^ proposed combining consistency, focusing, and defocusing clues in a four-dimensional polar plane image to optimize the depth map by utilizing complementary information provided by each other, in response to the blurring of the corresponding area caused by the occlusion of the focusing sequence and the corresponding changes in the focusing degree. Park et al.^6^ proposed an adaptive constrained defocus matching method, which divides the original focusing sequence into different image blocks and selects the unobstructed parts for defocus degree calculation to eliminate the influence of occlusion. Strecke et al.^7^ proposed to calculate the symmetry of refocused sequences by using views from four directions: up, down, left, and right, in order to address occlusion in depth extraction based on focused sequences.Suzuki et al.^8^ solved the problem of the limited range of optical field disparity. The principle of calculating depth based on the optimal slope of linear structures in Epipolar Plane Image (EPI) is that when fixing one dimension of the image plane coordinates and camera plane coordinates in a light field camera, the corresponding pixels are stacked in the perspective direction. Pixels located in different perspectives form a straight line, and the slope of the line reflects the depth information at the corresponding point. Depth can be estimated directly by calculating the slope of the line. Wanner et al.^9^ first proposed using structural tensors to estimate the slope of the oblique line in polar plane images, and then integrated local depth using fast denoising and global optimization methods. Li et al.^10^ proposed a new approach to reconstruct continuous depth maps using light fields, obtaining dense and relatively reliable local estimates from the structural information of densely sampled light field views, and then proposing an optimization method based on conjugate gradient method for iteratively solving sparse linear systems. Chen et al.^11^ focused on regularizing the initial label confidence map and edge strength weights, detecting partially occluded boundary regions through superpixel based regularization, and then applying a series of shrinkage and reinforcement operations on the label confidence map and edge strength weights of these regions to improve the accuracy of depth estimation in the presence of occlusion. Zhang et al.^12^ proposed a Spinning Parallelogram Operator (SPO) based on the assumption of maximizing the two regions of the epipolar line. By comparing the weighted histogram distance differences between the two regions of the epipolar line, the direction of the straight line is fitted. This method has good robustness for some weak occlusion situations. Sheng et al.^13^ further proposed a strategy for extracting polar plane images in all available directions based on SPO, and designed a depth information estimation framework that combines local depth and edge direction. Williem et al.^14^ used corner blocks and refocused images to measure the constrained angular entropy cost and the constrained adaptive defocus cost, and then combined these two new data costs to reduce the impact of occlusion. The depth estimation algorithm based on polar plane images is prone to interference from occlusion, noise, and other environments, and it requires a large amount of computation, usually requiring subsequent complex optimization to obtain a smoother depth map. Compared to traditional methods, deep learning-based methods have strong feature extraction and representation processing capabilities and use multi-layer neural networks to extract deep clues from light field data and generate depth values. These networks utilize the linear structural features of EPI or the correlation features of sub-aperture images to obtain the depth of the corresponding scene. For instance, Guo et al.^15^ designed an occlusion perception network to estimate the depth of light field images and optimize occlusion edges. Shi et al.^16^ used an optical flow network to obtain the initial depth map of the light field and optimized the depth using the hourglass network structure. Yoon et al.^17^ introduced the light field Convolutional neural network (LFCNN) to improve the angle and spatial resolution of the light field. However, most of the existing neural network-based light field depth estimation methods use branch weight sharing and end-to-end training for the entire network, thus failing to fully utilize the consistency and complementarity of depth information in different directions of the light field data. At the same time, the robustness of neural network logarithmic data is insufficient.

For the focus stack based digital refocusing method of light field, the more times a light field image is refocused, the more accurate the result will be obtained, but the more time it takes to refocus, this article mainly develops a depth calibration method based on the principle of light field imaging and theoretically analyzes the depth resolution. A method for identifying the optimal refocusing coefficient based on the Gaussian fitting is also proposed to reduce calibration and measurement time. Specifically, we built a microlight field imaging system, conducted calibration experiments, and analyzed the impact of different numbers of calibration points on identifying the optimal refocusing coefficient and calibration curve fitting. This is important, as using dot calibration plates and luminescent micropores for depth calibration provides a depth recognition method for particle images that saves calibration time and improves calibration efficiency. At the same time, from the perspective of fitting principles, reducing the number of refocuses will not decrease the robustness to noises. The method is an optimization and improvement of the refocusing method based on the light field focus stack. This method is equally effective in scenarios with occlusion.

## Measurement principles

### Principles and sampling of light field imaging

**Figure 1:**
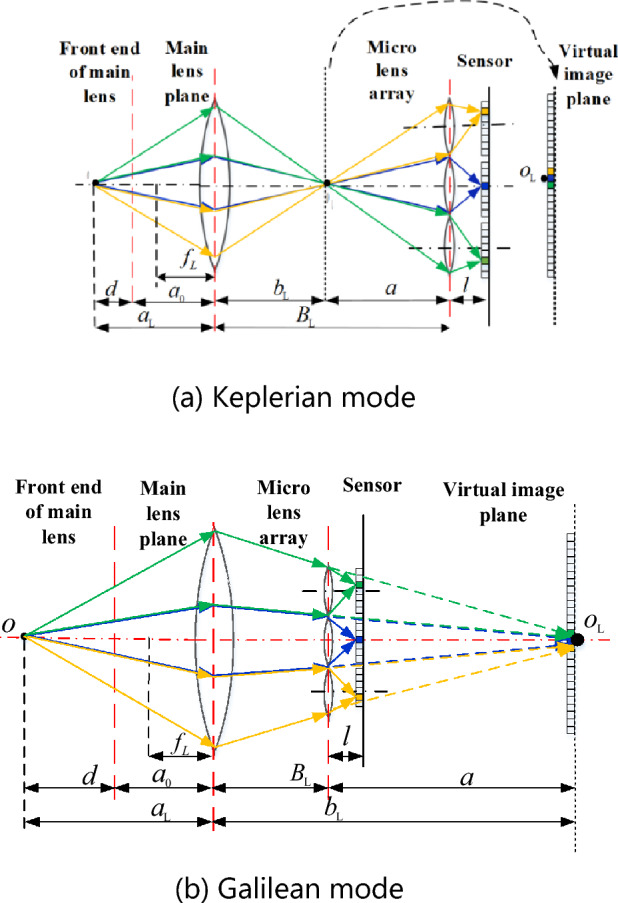
Schematic diagram of light transmission sampling for focusing light field camera.

Figure 1 illustrates the light transmission of two kinds of focused light field cameras: Keplerian and Galilean, where $a$ and *l* are the distance from the microlens plane to the virtual imaging plane of the main lens and the sensor plane, respectively. ${f}_{L}$ is the focal length of the main lens, and ${B}_{L}\left(=a+{b}_{L}\right)$ is the distance from the main plane of the main lens to the microlens. ${a}_{L}\left(={a}_{0}+d\right)$ is the distance between the distance between the position of interest in the object to the plane of the main lens, where ${a}_{0}$ is the distance from the front end of the main lens (lens group) to the main plane of the main lens, and *d* is the object's depth mentioned in this article*.*

The imaging detector of the unfocussed light field camera^18^ is located at one time the focal length ${f}_{m}$ of the microlens, and the main lens and imaging sensor are conjugated with respect to the microlens. The virtual image plane of the main lens and the imaging sensor plane in Fig. 1 are conjugated concerning the microlens. When the distance *l* from the microlens to the imaging detector is 1 ~ 1.5 times the microlens focal length, it is a Keplerian light field camera. Accordingly, it is a Galilean light field camera when the distance l from the microlens to the imaging detector is 0.5 ~ 1 times the focal length^19^. Assuming that along the optical axis of the main lens, the direction from the object point to the imaging detector is the positive direction. In the Kepler-type light field camera, the distance *a* from the virtual image plane to the microlens is positive, and the image on the detector is inverted. In the Galileo light field camera, the distance *a* from the virtual image plane to the microlens is negative, and the image on the detector is positive^20^. As depicted in Fig. 1, in the focused light field camera, the object point *o* is imaged on the virtual image plane via the main lens to the virtual image point ${o}_{L}$. The microlenses image the virtual image plane on the imaging sensor plane. The imaging detector of traditional two-dimensional cameras is located at the virtual image plane, where the virtual image point ${o}_{L}$ occupies N_T_ pixels (nine pixels in the figure), all with spatial information of the object points. The virtual image point ${o}_{L}$ via the N_M_ microlenses (presented in Fig. 1) are imaged on the imaging detector, making its spatial resolution $\frac{{N}_{T}}{{N}_{M}}$ times that of traditional 2D cameras. This increases the angle information by N_M_, thus sacrificing spatial information in exchange for angle information. In a light field camera, each microlens images the main lens to form a macro pixel, and several pixels at the same position relative to the center of the microlens are spliced to form a single view image according to the position sequence of the microlens in the sensor^21^. As depicted in Fig. 1, the pixels of each color are spliced to form a single view image, and the number of single view sampling pixels in each macro pixel of the focused light field camera exceeds 1. Figure 1 aims to enhance the reader's understanding; thus, only one pixel is drawn from a single perspective under each microscope.

### Refocus of light field

The light field can be parameterized by light rays and two parallel planes intersecting in space^22^. Let $L\left(u,v,x,y\right)$ represent the Radiant intensity of the beam passing through the point (*u*, *v*) on the plane where the microlens is located and the point (*x*, *y*) on the plane where the detector is located. Then the total energy $I\left(x,y\right)$ from the beam $L\left(u,v,x,y\right)$ received by the point (*x*, *y*) is:

1 $$I\left(x,y\right)=\iint L\left(u,\nu ,x,y\right)dudv$$

**Figure 2:**
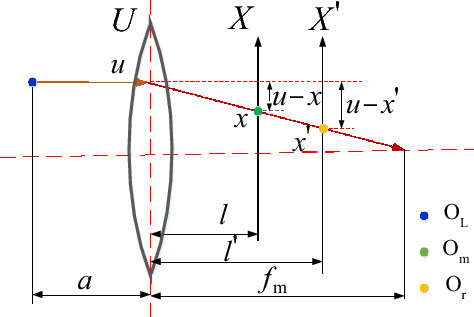
Schematic diagram of light field refocusing.

According to^23^, refocusing involves extracting a four-dimensional light field from the original two-dimensional light field image and reprojecting it onto a new imaging surface to obtain two-dimensional images at different plane positions. The refocusing principle diagram of a simplified two-dimensional light field is presented in Fig. 2.

A virtual image point ${o}_{L}$ is imaged as ${o}_{m}$ on the sensor through a microlens. ${o}_{r}$ is ${o}_{m}$, which is the point after refocusing transformation. *u*, *x*, and ${x}{\prime}$ are the coordinates of the intersection point between the beam *L* and the microlens plane *U*, the imaging detector plane *X*, and the refocusing plane ${X}{\prime}$, respectively. ${l}{\prime}$ is the distance between the microlens plane and the refocusing plane ${X}{\prime}$ and ${l}{\prime}=\alpha l$, where $\mathrm{\alpha }$ is the refocusing coefficient. From the similarity principle and coordinate relationship, it can be obtained that:

2 $$x=\frac{1}{\alpha }{x}{\prime}+\left(1-\frac{1}{\alpha }\right)u$$

Similarly, in the other two dimensions of the four-dimensional light field, the relationship between the coordinates of the intersection point ${y}{\prime}$ of the beam and the refocusing plane ${Y}{\prime}$ and the coordinates $y$ of the intersection point of the beam and the detector plane are:

3 $$y=\frac{1}{\alpha }{y}{\prime}+\left(1-\frac{1}{\alpha }\right)v$$

Combining Eqs. (2), (3), and (1) provides the refocusing formula:

4 $$I\left({x}{\prime},{y}{\prime}\right)=\iint L\left(u,v,\frac{1}{\alpha }{x}{\prime}+\left(1-\frac{1}{\alpha }\right)u,\frac{1}{\alpha }{y}{\prime}+\left(1-\frac{1}{\alpha }\right)v\right)dudv$$

### Depth measurement and depth resolution based on refocusing

#### Principle of depth measurement

According to the Gaussian imaging formula, it can be concluded that:

5 $$\frac{1}{a}+\frac{1}{{\alpha }_{opt}l}=\frac{1}{{f}_{m}}$$

6 $$\frac{1}{{a}_{L}}+\frac{1}{{b}_{L}}=\frac{1}{{f}_{L}}$$

Parameter ${\alpha }_{opt}$ is the optimal refocusing coefficient corresponding to the refocusing plane at the clearest position of the object point imaging. The relationship between depth *d* and the optimal refocusing coefficient is obtained by combining Fig. 1 and 2 with the Gaussian optics formula:

7 $$d={\left\{{f}_{L}^{-1}-{\left[{B}_{L}-{\left({f}_{m}^{-1}-{\left({\alpha }_{opt}l\right)}^{-1}\right)}^{-1}\right]}^{-1}\right\}}^{-1}-{a}_{0}$$

Formula (7) is organized as follows:

8 $$d=\frac{{c}_{2}{\alpha }_{opt}+{c}_{0}}{1-{c}_{1}{\alpha }_{opt}}$$

Among them:

9 $${c}_{0}=\frac{{f}_{m}{B}_{L}{a}_{0}-{f}_{m}{B}_{L}{f}_{L}-{f}_{m}{f}_{L}{a}_{0}}{{f}_{m}{f}_{L}-{f}_{m}{B}_{L}}$$

10 $${c}_{1}=\frac{l{f}_{m}+l{f}_{L}-l{B}_{L}}{{f}_{m}{f}_{L}-{f}_{m}{B}_{L}}$$

11 $${c}_{2}=\frac{l{B}_{L}{f}_{L}-l{f}_{m}{f}_{L}-l{B}_{L}{a}_{0}+l{f}_{m}{a}_{0}+l{f}_{L}{a}_{0}}{{f}_{m}{f}_{L}-{f}_{m}{B}_{L}}$$

where the coefficients ${c}_{0}$, ${c}_{1}$, and ${c}_{2}$ depend only on the fixed parameters of the imaging system. Next, a detailed theoretical analysis will be conducted on the depth resolution of this method, and further research will be conducted on the optimal refocusing coefficient for each depth position.

#### Depth resolution

In order to analyze the depth resolution, based on formula (8), taking the derivative of d over ${\alpha }_{opt}$ yields:

12 $$\frac{{\text{d}}d}{{\text{d}}{\alpha }_{opt}}=\frac{{c}_{2}+{c}_{0}{c}_{1}}{{\left(1-{c}_{1}{\alpha }_{opt}\right)}^{2}}$$

By organizing formula (8) and bringing it into formula (12), it can be concluded that:

13 $$\frac{{\text{d}}d}{{\text{d}}{\alpha }_{opt}}=\frac{{\left({c}_{1}d+{c}_{2}\right)}^{2}}{{c}_{2}+{c}_{1}{c}_{0}}$$

We set the depth resolution of a certain depth position *d* to $\Delta d$. The increment of the optimal refocusing coefficient at the corresponding depth position is $\Delta {\alpha }_{opt}$. Thus:

14 $$\Delta d=\frac{{\left({c}_{1}d+{c}_{2}\right)}^{2}}{{c}_{2}+{c}_{1}{c}_{0}}\Delta {\alpha }_{opt}$$

From the previous analysis, ${c}_{0}$, ${c}_{1}$, and ${c}_{2}$ are the coefficients comprising the fixed parameters of the imaging system. So, formula (14) infers that the depth resolution $\Delta d$ and depth position *d* are related to the increment of the optimal refocusing coefficient, which can be resolved at this depth position $\Delta {\alpha }_{opt}$. Note that $\Delta {\alpha }_{opt}$ is related to adjacent refocused images during image processing. Hence, in the same batch of image processing, $\Delta {\alpha }_{opt}$ can be considered a fixed value, and thus, the depth resolution varies with the depth position. The minimum depth resolution is particularly important for depth resolution. From formula (14), it is known that $\frac{\Delta {\alpha }_{opt}}{{c}_{2}+{c}_{1}{c}_{0}}>0$. Therefore, formula (14) is a parabolic curve with an upward opening, and the depth is ${d}_{ms}$ when the depth resolution reaches the minimum value. Then:

15 $${d}_{ms}=-\frac{{c}_{2}}{{c}_{1}}$$

By incorporating formulas (10) and (11) into formula (15), it can be concluded that:

16 $${d}_{ms}=\frac{{f}_{L}\left({f}_{m}-{B}_{L}\right)}{{f}_{m}+{f}_{L}-{B}_{L}}-{a}_{0}$$

## Experiment and processing

### Qualitative verification of the rendering effect of refocused light field images

**Figure 3:**
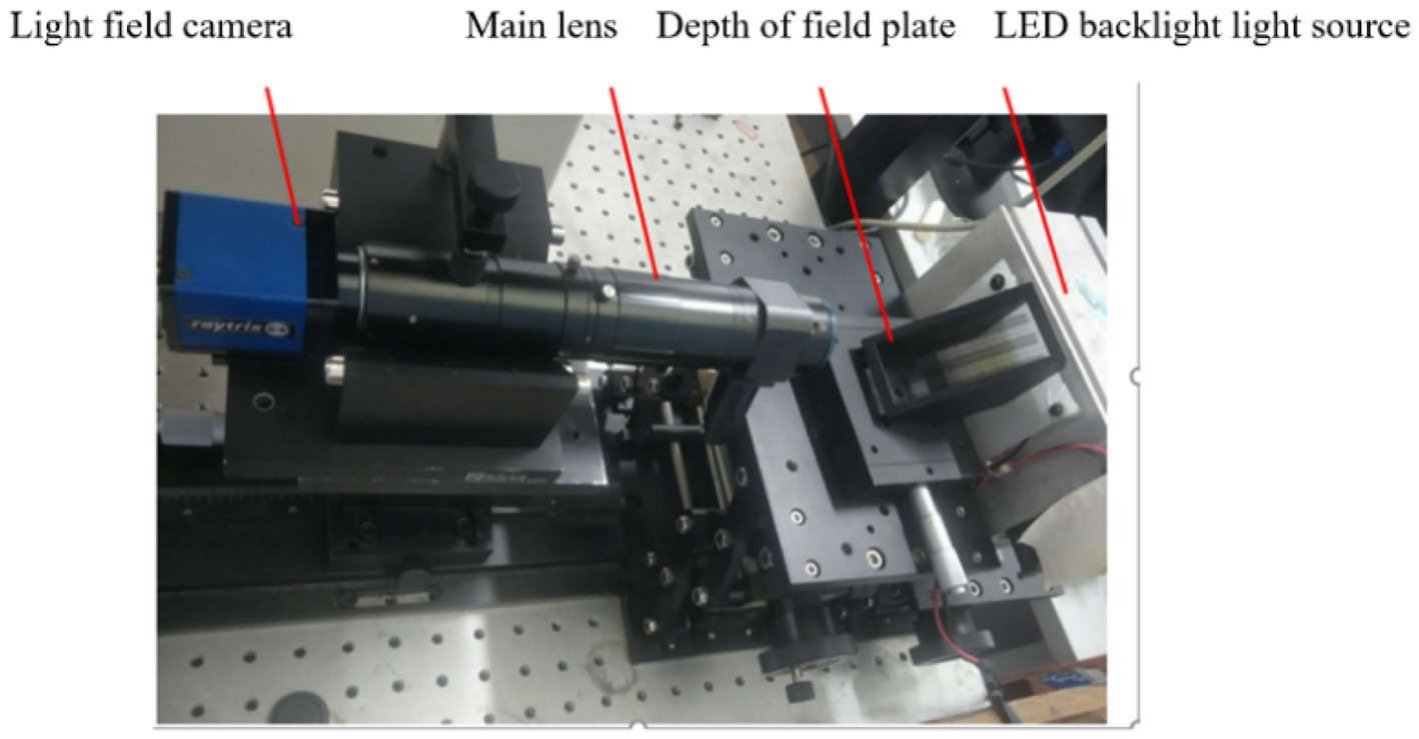
Photo of the experimental setup for qualitative verification of the refocusing effect.

To qualitatively verify the relationship between the refocusing coefficient *α* and depth *d* and demonstrate the effect of refocusing light field rendering, we experimented using the qualitative verification experimental device for the refocusing effect shown in Fig. 3. This setup uses a LED backlight, the depth of field target is photographed with a Raytrix R12 Micro light field camera, and the VSZ-0745CO lens is configured. The aperture and magnification of this lens can be adjusted. For the experimental conditions, the camera's exposure time is 20 ms, the magnification is 2.74, and the F-number is 26, which is equal to the F-number of the Raytrix R12 Micro light field camera.

**Figure 4:**
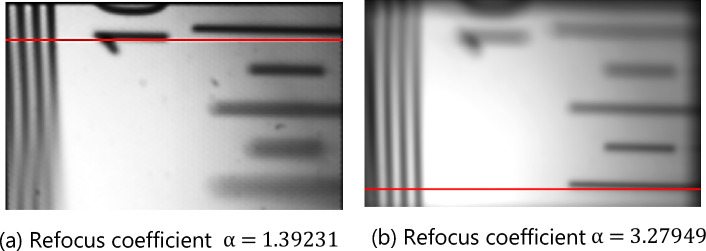
Refocus image of depth plate focusing on different depth planes.

Figure 4a and b illustrate the refocusing results obtained from the refocusing formula for α = 1.39231 and α = 3.27949.

**Figure 5:**
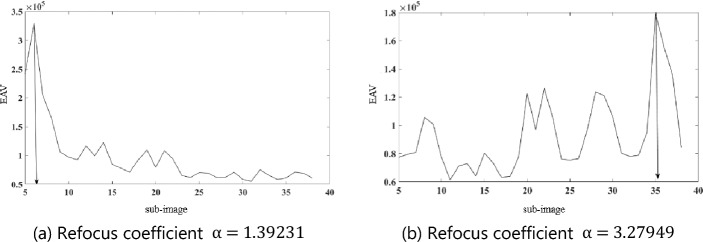
Sharpness map of different regions of the refocused image along the vertical direction.

The refocused images in Fig. 4a and b were divided into 38 sub-images along the vertical direction. The sharpness of the pixels was characterized based on the point sharpness function Edge Acutance Value (EAV) gradient^24^, and the sum of the sharpness of each sub-image was calculated. The obtained sharpness curve of the target refocused image along the vertical direction is presented in Fig. 5a and b, with the abscissa being the number of sub-images.

When the imaging is the clearest, then $\mathrm{\alpha }={\alpha }_{opt}$. In the experiment, the upper edge of the depth of the field target is relatively far from the lens. That is, d is larger, and the lower edge is the reverse. When $\mathrm{\alpha }=1.39231$, the clear position of the refocused image is near the upper edge, which is marked by the red line in Fig. 4a. When $\mathrm{\alpha }=3.27949$, the clear position of the refocused image is near the lower edge, which is marked by the red line in Fig. 4b and is consistent with Eq. (8).

**Figure 6:**
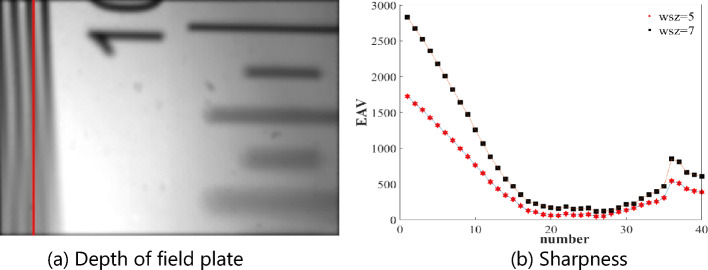
Sharpness map of different areas along the vertical direction in the stripe area of the depth plate refocused image.

Figure 6b shows the stripe lines (i.e., red vertical lines) at the edge of the depth plate in Fig. 6a, with a width of 5 and 7 pixels, respectively, divided into 40 parts vertically. The clarity of each image region is calculated using the EAV gradient to represent the sharpness of each pixel. Since the side of the depth of field plate is a right-angled triangle, the lower edge surface is closer to the lens, i.e., d is smaller, and the upper edge surface is further away from the lens, i.e., d is larger. Referring to the inverse relationship between the optimal refocusing coefficient α_opt_ and *d* in the formula in Section 2.3, the experimental results are as follows: The sum of the sharpness for each segment is plotted on the graph, which reveals that the clarity gradually decreases as we move from the upper edge toward the center region of the image. This finding is consistent with the theoretical expectations. From the center region to the lower edge of the depth of the field plate, the sharpness slowly increases, which can be attributed to the image extending beyond the measurement range of the system.

### Deepth calibration experiment

**Figure 7:**
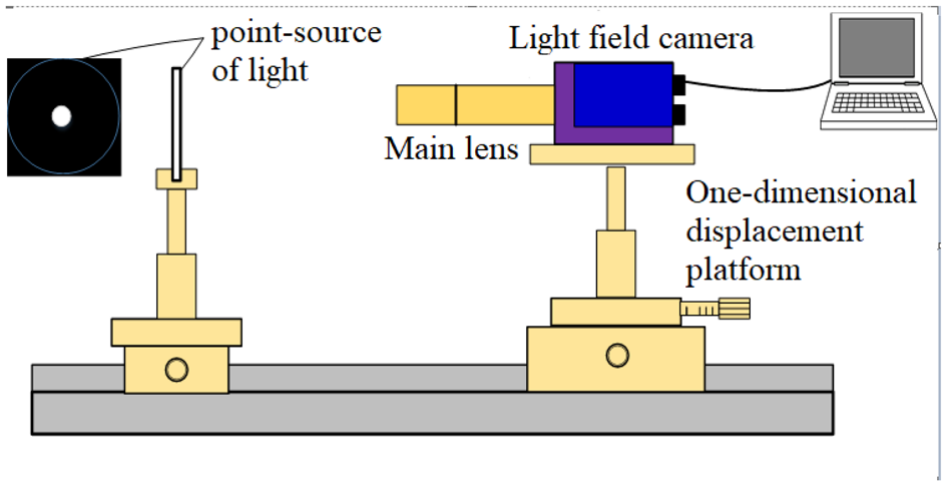
Schematic diagram of the point light source depth calibration experimental device.

Figure 7 depicts the experimental system setup for depth calibration using a point light source. The experimental system comprises a Raytrix R12 light field camera, a VSZ-0745CO main lens, a one-dimensional displacement platform, and an electric guide rail. The imaging object is an LED point light source with an aperture diaphragm diameter of 5 microns.

**Figure 8:**
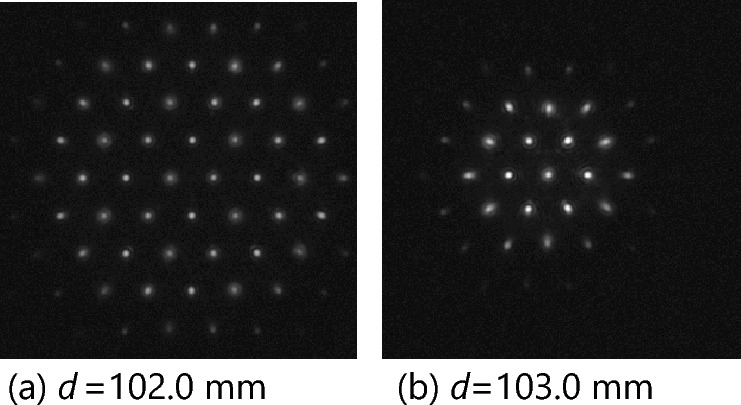
Partial enlarged image of the original light field of a point light source (0.344 mm × 0.344 mm).

Using an electric displacement platform, we gradually move the point light source from a distance of 99.0 mm from the front end face of the lens to 104.5 mm from the front end face in steps of 0.1 mm. Then, we extract the four-dimensional light field information in the five original light field images captured at each position and then take the average to obtain the average light field image at that position for subsequent processing to reduce noise impact. This process provides 56 groups of images. Figure 8 illustrates a partially enlarged image of the original light field of a point light source at different depths.

The image standard deviation *δ* is used to represent image clarity, and 500 refocusing images are formed at equal intervals within $\alpha \in \left(\mathrm{0.1,5}\right)$. Comparing the clarity among the 500 refocusing images, the value of α that provides the image with the highest clarity is ${\alpha }_{opt}$, corresponding to depth *d*. In response to the time-consuming problem of creating many refocused images during depth calibration, this paper conducts fewer refocusing operations on the light field images at each depth position. Each refocused image corresponds to a refocusing coefficient $\mathrm{\alpha }$, and then the clarity of each refocused image is calculated. A Gaussian function, which describes the imaging property and quality of the optical imaging system, is used to calculate the refocusing coefficient α that fits with the sharpness of the refocused image^25,26^. The value of $\mathrm{\alpha }$ that corresponds to the sharpness peak of the Gaussian function obtained after fitting is the ${\alpha }_{opt}$, which corresponds to depth *d.*

17 $$\mathrm{\alpha }\left(\delta \right)=\frac{1}{\sqrt{2\pi \sigma }}exp\left(-\frac{{\left(\delta -\upmu \right)}^{2}}{2{\sigma }^{2}}\right)$$

where $\upsigma$ is the standard deviation, and $\upmu$ is the mathematical expectation.

## Analysis of calibration results

### Results of calibrating the optimal refocusing coefficient with fewer points

**Figure 9:**
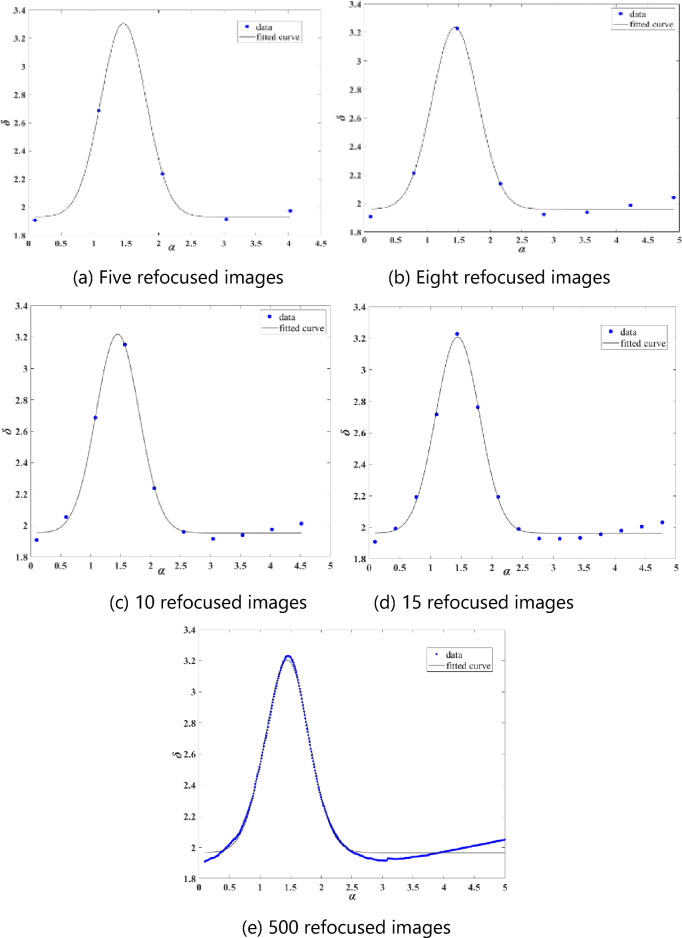
d = 103.6 mm α–δ chart.

Figure 9 shows the α–δ curve after Gaussian fitting with 5, 8, 10, 15, and 500 refocusing images when *d* = 103.6 mm. It can be seen from Fig. 9 that the peak value of *δ* at this depth position is around 3.2, and the corresponding optimal refocusing coefficients ${\alpha }_{opt}$ are all around 1.5. When five refocusing images are used, the peak value of *δ* differs more compared to using more or fewer images, but it is still around 3.2 and ${\alpha }_{opt}$ is also around 1.5. The optimal refocusing coefficient without Gaussian fitting is also around 1.5, proving our method’s feasibility.

**Figure 10:**
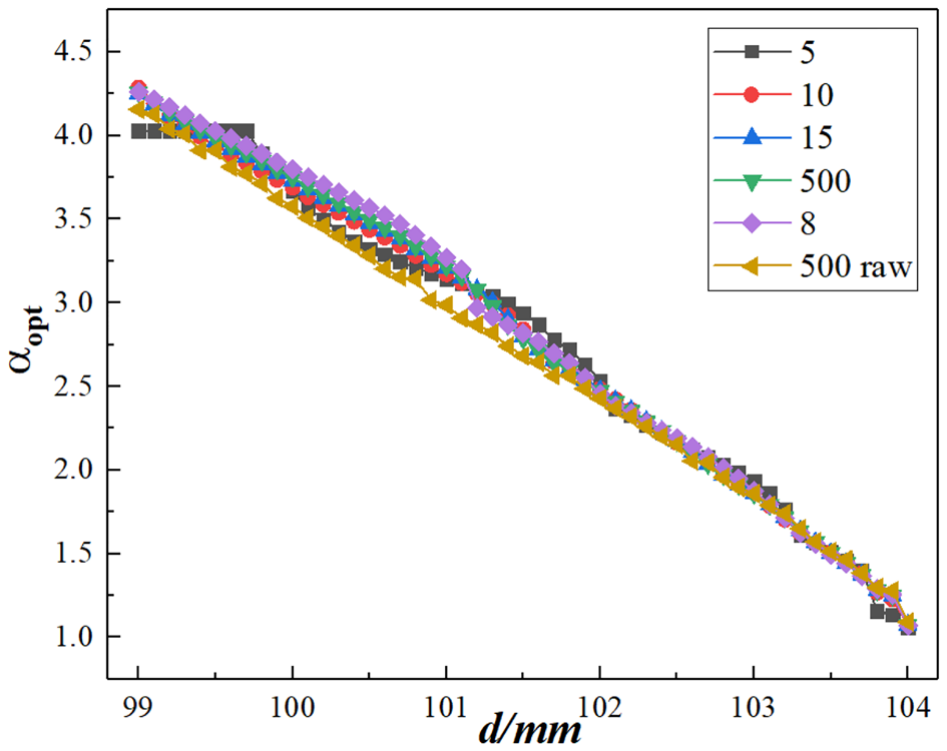
5 micron point light source *d*~${\alpha }_{opt}$ diagram.

Figure 10 illustrates the results of calibrating each depth position with a 5-micron point light source. The graph represents the relationship between each depth value *d*, and its corresponding optimized alpha ${\alpha }_{opt}$. The numbers 5, 8, 10, 15, and 500 denote the number of refocusing performed at each depth position. The resulting curve is obtained by Gaussian fitting $\mathrm{\alpha }$ and $\delta$, where the $\mathrm{\alpha }$ value that corresponds to the maximum value of $\delta$ is ${\alpha }_{opt}$. Figure 9 highlights that when performing five refocusing at each depth position and using Gaussian fitting to determine ${\alpha }_{opt}$, a deviation occurs around the depth range of 99–100 mm. The results are better aligned using 8, 10, 15, and 500 refocusing.

Therefore, when using the Gaussian fitting method to determine ${\alpha }_{opt}$ it is important to consider the influence of the number of refocusing images on the depth calibration range. This study suggests selecting around 10 refocusing images per depth position to determine ${\alpha }_{opt}$, which reduces the time required for depth calibration significantly. Indeed, only 1/50 of the time is required when using 500 refocusing iterations.

### Analysis of depth results and depth resolution for less point calibration

**Figure 11:**
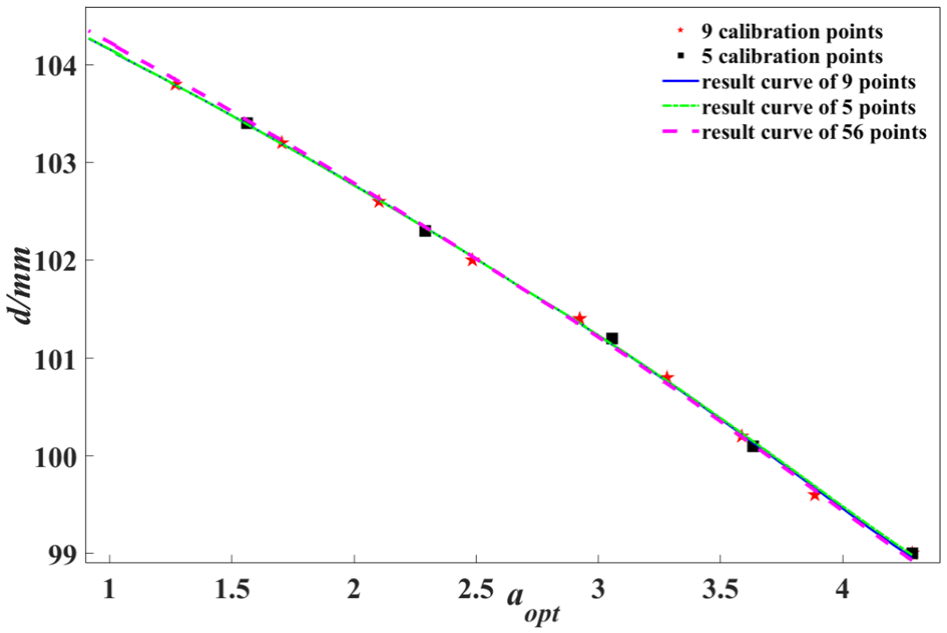
Depth calibration curve.

Figure 11 illustrates the depth calibration results obtained by selecting the optimal refocusing coefficient using 10 refocusing images. The rectangular and pentagonal markers represent the 5 and 9 data points used during calibration. The solid line, dashed line, and dotted line correspond to the results obtained from calibration using 5, 9, and 56 points, respectively. Table 1 reports the values of ${c}_{0}$, ${c}_{1}$, and ${c}_{2}$ associated with the indicated points.

**Table 1:** Depth calibration results.

Coefficient	5 points	9 points	56 points
*c*_0_	105.4044	105.3868	105.5332
*c*_1_	0.0476	0.0509	0.0447
*c*_2_	− 6.2068	− 6.5411	− 5.9700

In Eq. (14), the values of the denominator ${c}_{2}+{c}_{1}{c}_{0}$ are − 1.18955, − 1.17691, and − 1.25267, respectively. Additionally, it is worth noting that all values of ${c}_{1}$ are greater than 0, while all values of ${c}_{2}$ are less than 0. This indicates that the depth resolution of the system used in this study decreases as depth increases.

## Conclusion

This paper describes the image rendering method for refocusing by combining ray tracing and integral imaging principles. We apply this method to capture and process images of a depth-of-field chart, with the results demonstrating that the refocused images agree well with the theoretical analysis. Furthermore, the experimental methodology for depth calibration based on the light field imaging theory model is improved. We showcase the *α–δ* curve obtained by Gaussian fitting and quantitatively select the optimal refocusing coefficient using different numbers of refocusing images at specific depths and the corresponding *d* ~ *α*_opt_ curve within the measurement range. The results indicate that when using Gaussian fitting to determine ${\alpha }_{opt}$ The applicable range is limited with a few refocusing images, e.g., five images examined in this paper. It is found that selecting around 10 refocusing images is preferable, significantly reducing the processing time required for image handling during the depth calibration process. Moreover, from the perspective of imaging principles combined with image processing, a detailed theoretical analysis of the depth resolution of this depth measurement method is conducted. For the specific light field system used in this study, the numerical value of the depth resolution decreases with increasing depth and increasing optimal refocusing coefficient.

## Data Availability

Data sets generated during the current study are available from the corresponding author on reasonable request.
